# Bonding Behavior of Deformed Steel Rebars in Sustainable Concrete Containing both Fine and Coarse Recycled Aggregates

**DOI:** 10.3390/ma10091082

**Published:** 2017-09-14

**Authors:** Sun-Woo Kim, Wan-Shin Park, Young-Il Jang, Seok-Joon Jang, Hyun-Do Yun

**Affiliations:** 1 Department of Construction Engineering Education, Chungnam National University, Daejeon 34134, Korea; sw.kim@cnu.ac.kr (S.-W.K.); salshin@cnu.ac.kr (W.-S.P.); jang1001@cnu.ac.kr (Y.-I.J.); 2 Department of Architectural Engineering, Chungnam National University, Daejeon 34134, Korea; jang@cnu.ac.kr

**Keywords:** recycled aggregate concrete, pull-out test, bond strength, slip, casting position effect

## Abstract

In order to assess the bond behavior of deformed steel rebars in recycled-aggregate concrete (RAC) incorporating both fine and coarse recycled aggregate, pull-out tests were carried out in this study on 16-mm diameter deformed steel rebars embedded concentrically in RAC. The concrete was designed using equivalently mixed proportions of both recycled coarse aggregate and recycled fine aggregate. The tests employed five types of recycled aggregate replacement combinations and three types of rebar placement orientation (i.e., vertical bars and two-tiered and three-tiered horizontal bars). Based on the pull-out test results, the maximum bond strength tended to decrease and the slip at the maximum bond strength increased as the average water absorption of the aggregate increased, irrespective of the rebar orientation or placement location within the concrete member. The pull-out test results for the horizontal steel rebars embedded in RAC indicate that the casting position effect could be determined from the mid-depth of the concrete member, irrespective of the member’s height. The normalized bond versus slip relationship between the deformed rebar and the RAC could be predicted using an empirical model based on regression analysis of the experimental data.

## 1. Introduction

The construction industry is resource intensive and often relies on extensive use of natural resources. Furthermore, the demolition of old buildings creates waste products, which in turn puts enormous pressure on the environment to cope with the disposal of the resultant byproducts. For example, in Korea, numerous old apartments buildings built in the 1970s have been demolished, and the difficulties associated with the disposal of the construction waste have caused major environmental problems, including soil/water pollution issues. In order to help solve this problem, the Korean government enacted a law [1] in 2003 that requires construction waste to be recycled. According to statistical data from the Korean Ministry of Environment for 2012 [2], 187,000 tons (48.9% of total waste) per day of construction waste were generated, of which 84.4% were recycled. In 2005, the Recycled Aggregate Quality Standard [3] was enacted so that the material and structural performance of concrete containing recycled aggregate could be studied and standardized.

**Figure 1:**
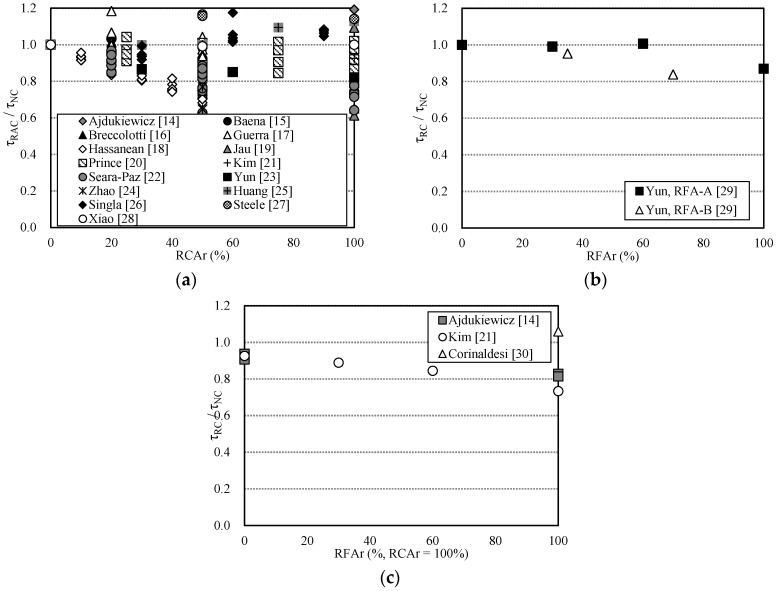
Comparison of bond strength ratio (*τ*_RAC_/*τ*_NC_) with recycled aggregate. (**a**) Recycled coarse aggregate (RCA) concrete; (**b**) Recycled fine aggregate (RFA) concrete; (**c**) Both recycled fine and coarse aggregate (RFCA) concrete.

The main components of concrete mixtures are coarse and fine aggregates, which comprise about 70% of the total concrete weight. Due to this high percentage of aggregate in concrete, a sustainable and economical source of high quality aggregate is essential to the concrete industry. However, most of the previous studies on this topic examined recycled-aggregate concrete (RAC) produced using a mixer in a laboratory [4,5,6,7,8]. Therefore, the results so far obtained can hardly be applied to practical construction with ready-mixed concretes because the investigations are based on laboratory, not field, results. In addition, compared to the number of studies on recycled coarse aggregate (RCA), research on concrete with recycled fine aggregate (RFA) [9,10,11,12,13] is scant due to the difficulty in producing high-quality fine aggregates. Previous studies that investigated the mechanical properties of RFA concrete found that the compressive strength, the tensile strength, and the elastic modulus were slightly low, but still acceptable, at least for up to 30% replacement level. Figure 1 represents the bond strength ratios of RAC to normal concrete, and the normalized bond strength ratios for RCA, RFA, and RFCA are shown in Figure 1a–c, respectively. For steel rebars embedded in concrete with RCA, most of the pull-out test results from other research efforts [14,15,16,17,18,19,20,21,22,23,24] indicate that the bond strength decreases as the RCA replacement level (RCA*r*) increases, whereas some other test results [25,26,27,28] suggest that the bond strength slightly increases as the RCA*r* increases. In the case of concrete with RFA, the characteristics of bond-strength degradation depend upon the quality of RFA, especially in terms of water absorptivity [29]. In terms of the bond strength-slip relationship between deformed steel rebar and RAC, some studies [14,15,16,17,18,19,20,21,22,23,24,25,26,27,28,29,30] report that RAC exhibits bond strength that is similar to that of normal concrete, at least for up to 30% replacement, regardless of whether RCA or RFA is used. However, studies for concrete incorporating both recycled fine and coarse aggregate (RFCA) [14,21,30] investigate the effect of RFA replacement levels (RFA*r*) only; no natural coarse aggregate is used. The pull-out tests indicate that the bond strength decreases as the RFA*r* increases.

As shown in Figure 1, regardless of whether RCA or RFA is used, the 100% replacement level is not recommended for application to the mix design of concrete because the concrete could not meet the requirement for both the development and splice length of bars. Hence, in order to use RCA and/or RFA in concrete, the upper limit should be introduced for the replacement level.

Therefore this paper investigates the bonding behavior between deformed steel rebars and concrete containing 75% replacement of a blend of RCA and RFA (i.e., RCA + RFA). The recycled aggregate used in this study was obtained from the demolition waste of old apartments that were constructed originally with normal strength (21 MPa) concrete. For concrete members that were cast parallel to the steel reinforcement, experimental bond strength levels derived from pull-out tests were compared with calculated values proposed by Orangun [31], Darwin [32], the Comité Euro-International du Béton, and the Fédération Internationale de la Précontrainte (CEB-FIP) [33]. In addition, the steel-concrete bond strength at various concrete depths (75 mm, 225 mm, and 375 mm) was considered in order to investigate the casting position effect coefficient suggested by the American Concrete Institute (ACI) [34] and Canadian Standards Association (CSA) code provisions [35]. In order to describe the horizontally-placed steel reinforcement, the concrete was cast in a perpendicular direction to the steel reinforcement. The aims of this study were to investigate the comparative effects of both RCA and RFA on bond behavior of deformed steel rebars and ready-mixed RAC, and to improve the practical use of RAC in various fields.

## 2. Experimental Program

### 2.1. Materials

#### 2.1.1. Aggregate

**Figure 2:**
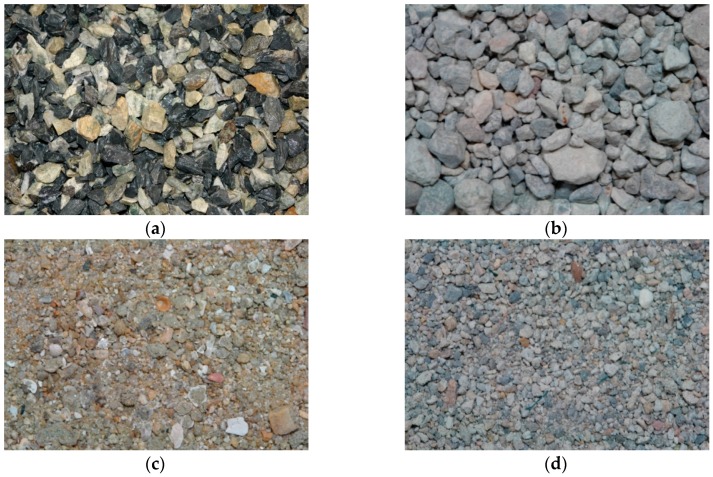
Aggregate used in this study. (**a**) Natural coarse aggregate (NCA); (**b**) RCA; (**c**) Natural fine aggregate (NFA); (**d**) RFA.

The RCA and RFA used in this study were obtained from the demolition of apartment buildings in Korea and were produced by crushing the waste concrete using a jaw crusher. The compressive strength of the original waste concrete was 21 MPa. Crushed stone and river sand were respectively used for natural coarse aggregate (NCA) and fine aggregate (NFA). The maximum grain size of both natural and recycled coarse aggregates was 25 mm, in accordance with KS F 2527 [36]. Figure 2 shows the four types of aggregate used in this study, and Table 1 lists their physical characteristics.

#### 2.1.2. Binder

Common Portland cement (c) Type 1 conforming to Korean Standard (KS) L 5201 [36] was used as the binder in this study. The cement had a specific gravity of 3.15 g/cm^3^, a plain surface area of 3.06 cm^2^/g, and initial and final setting times of 3 h 35 min and 5 h 35 min, respectively.

#### 2.1.3. Mix Proportions

The designed mix proportions of the concrete specimens are listed in Table 2. The replacement levels of the RCA and RFA are termed as the ratio of the recycled aggregate to the total coarse or fine aggregate (by weight), respectively. In order to simulate actual concrete mixing conditions for construction in practice, all materials were added at the plant and mixed using ready-mix concrete trucks. Due to their high water absorption characteristics, RCA and RFA were presoaked prior to mixing in the ready-mix trucks. The amount of water used to presoak the recycled aggregate was calculated according to the effective absorption of the recycled aggregate. The main differences among the five groups were the RCA*r* and RFA*r*, which were 0%, 15%, 30%, 45%, and 60%. The total recycled-aggregate replacement levels, i.e., the sum of the RCA and RFA, were 0% and 75%.

#### 2.1.4. Steel Rebars

Deformed steel rebars with a diameter of 16 mm (D16) were used for this investigation. Tensile tests of the rebar were conducted for five tensile samples, and the D16 showed a yield strength of 422 MPa at 0.21% strain, an ultimate strength of 609 MPa, and an elastic modulus value of 198 GPa.

### 2.2. Pull-Out Specimen and Test Method

The bonding behavior of deformed rebar with a diameter of 16 mm (D16) in concrete was studied by conducting direct pull-out tests of deformed rebars embedded in normal concrete and in RAC specimens. In general, the bond strength of rebar with bigger diameter is larger than those with smaller diameters, and this size effect was examined in previous study [37].

As listed in Table 3, the recycled-aggregate replacement level was the main variable in this study (five levels). All specimens were fabricated according to ASTM C234 [38], and all specimens had the same anchored length (64 mm, 4*d_b_*) at the non-loading face, and concrete cover-to-bar diameter ratio (*C*/*d_b_* = 4) to prevent splitting failure of concrete.

To evaluate the effect of recycled aggregate on the bond behavior of deformed bars in concrete, three types of pull-out specimen were fabricated;

- V type: To evaluate bond behavior of vertical reinforcement in concrete, a deformed bar was vertically installed at the center of each specimen.
- H type: To evaluate bond behavior of horizontal reinforcement in concrete, deformed bars were horizontally installed at depths of 75 (HB), 225 mm (HT) from the bottom of the specimen.
- R type: To evaluate the experimental modification factor related to top-cast rebars and the bond slip in pull-out test specimens, deformed bars were horizontally installed at depths of 75 (RB), 225 (RM) and 375 mm (RT) from the bottom of the specimen.

**Figure 3:**
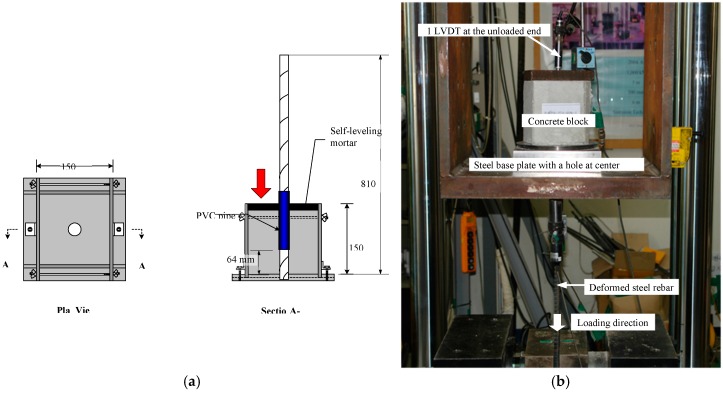
Pull-out test. (**a**) Typical test specimen (V-type); (**b**) Test set-up.

Cubic specimens measuring 150 mm × 150 mm × 150 mm were cast to embed a deformed bar; then, concrete was placed in the formwork. Refer to reference [29] for detailed descriptions and figures about size of specimen, casting and separating method. Pull-out test setup and measuring instrumentations are shown in Figure 3.

## 3. Results and Discussion

### 3.1. Mechanical Properties of Concrete

Twenty-five cylindrical specimens (D = 100 mm); 20 for compressive tests and five for tensile splitting tests for each mixture, were cast in steel molds and kept in a moist room at 23 °C and 95% relative humidity for 24 h until demolding. The specimens were then placed in water at 23 °C for a total curing period of 28 days. At 28 days after fabrication, compressive and splitting tensile tests were conducted in accordance with KS F 2405 and KS F 2423 [36], respectively.

The results of the compressive and splitting tensile tests are presented in Table 4; each value is the average of 20 and five test results for compressive and tensile splitting tests respectively. The compressive strength levels of the RAC were 13% to 16% higher than that of the normal concrete. For the RAC, no noticeable effects of the recycled aggregate on the compressive strength and elastic modulus values were evident. The tensile splitting strength values of the RAC specimens were 13% to 23% higher than those of the normal concrete.

### 3.2. Effect of Recycled-Aggregate Replacement Level on Bond Strength

For each recycled-aggregate replacement level, the bond strength *τ* was calculated as the stress between the rebar and the surrounding concrete along the embedded portion of the rebar, as follows:

(1) $$\tau =\frac{F}{\pi dl}$$

where *F* is the pull-out force of the deformed steel rebar; *l* is the bonded length; and *d* is the diameter of the bar.

**Figure 4:**
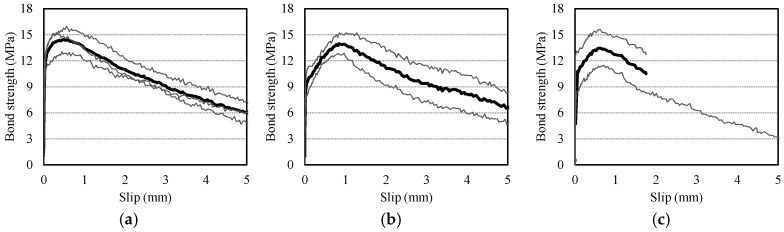

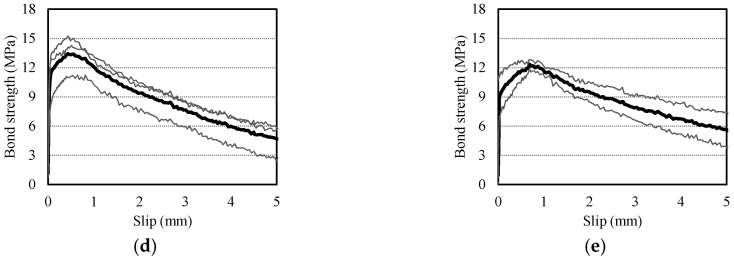
Bond strength-slip curves of V-type specimens. (**a**) G0F0V; (**b**) G15F60V; (**c**) G30F45V; (**d**) G45F30V; (**e**) G60F15F.

Figure 4 shows the bond strength-slip curves of the pull-out specimens; the bold line designates the mean curve. The curves demonstrate that the maximum bond strength (*τ*_0_) tends to decrease and the slip at the maximum bond strength (*s*_0_) increases as the RCA*r* increases. Bond behavior can be divided into four stages: (I) micro-slip, (II) internal cracking, (III) descending, and (IV) residual. For Stage I, whereas the G0F0 specimen had a high bearing stress that was about 90% of the peak stress, the G15F60V, G30F45V, G45F30V, and G60F15V specimens showed 71%, 77%, 85%, and 74%, respectively. For Stage II, among the RAC specimens, G30F45V and G45F30V showed relatively higher cracking stress levels and less slip than the other specimens. For Stages III and IV, no noticeable difference was evident among all the specimens, with similar stress levels around 6 MPa at the slip of 5 mm.

The bond strength obtained from the tests are summarized in Table 5. In the table, the pull-out test results are compared with the calculated bond strength levels suggested by Orangun [31], Darwin [32], and the CEB-FIP [33]. The measured bond strength exceed the calculated bond strength levels suggested by Orangun and Darwin, except for the G60F15 pull-out specimen.

According to the CEB-FIP model code [33] for monotonic loading, the bond strength (*τ*_0_) between the ribbed rebar and the surrounding concrete is defined as in Table 6.

**Figure 5:**
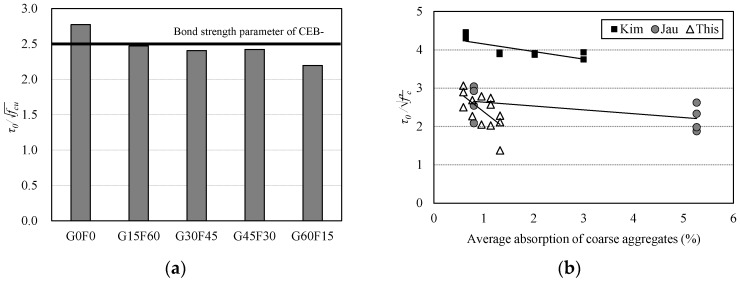
Normalized bond strength of pull-out specimens. (**a**) Relation with replacement level; (**b**) Relation with average absorption of coarse aggregate.

Figure 5 shows the bond strength levels of the test results normalized by compressive strength ($\sqrt{f_{cu}}$). The degradation of the bond strength can be seen from the normalized bond strength levels. As presented in Figure 5a, the normalized bond strength levels of the RAC are between approximately 88.8% and 98.8% of 2.50, which is the bond parameter in the CEB-FIP model code. Compared with the CEB-FIP specifications, all of the RAC pull-out test specimens showed lower bond strength levels, whereas the G0F0 specimen exhibited sufficient bond strength. Figure 5b shows that the normalized bond strength decreased as the average absorption of the coarse aggregate increased. It can be inferred then that bond strength was related to the quality of the specimen, especially the water absorption property of coarse aggregate that comprised between 30% and 40% of the total concrete volume. In addition to the water absorption properties, the bond strength was affected by the shape of the coarse aggregate, in particular the roundness of the RCA.

The bond behavior of RAC has been studied by many researchers. However, in most of the previous studies, relatively small coarse aggregate (<15 mm) was used, even though the size of coarse aggregate for practical construction was in the range of 20 mm to 25 mm. Therefore, among the pull-out results cited in the literature on RAC, only those where the maximum aggregate size was similar to the maximum aggregate size adopted in this study [19,23] were considered (see Table 7). In this study, the total absorption of the aggregate (RCA + RFA) for the RAC specimens was fixed to around 2.08 percent. So, the average absorption of the coarse aggregate was calculated in order to evaluate the effect of recycled aggregate on bond strength.

**Figure 6:**
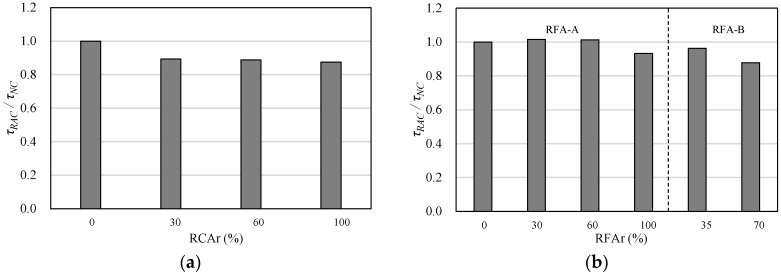

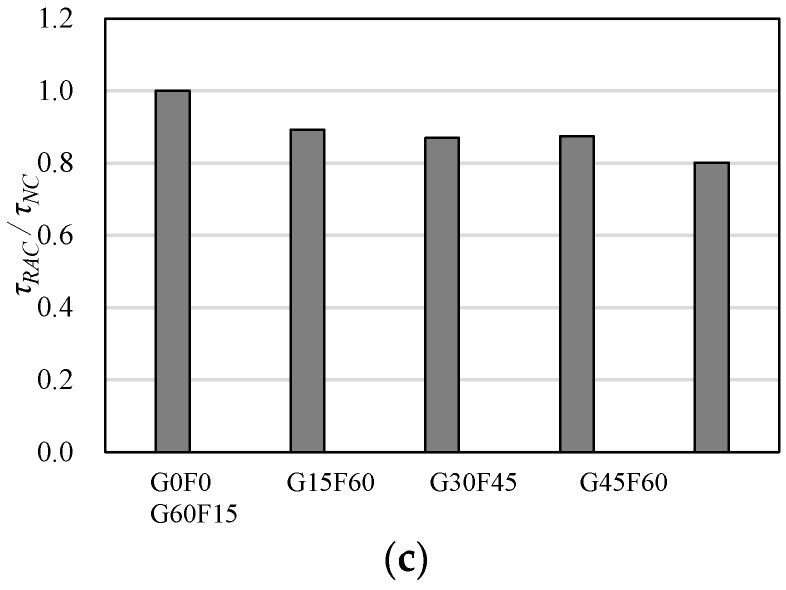
Comparison of bond strength ratio for pull-out test specimens. (**a**) RCA [23]; (**b**) RFA [29]; (**c**) RFCA.

For a comparison of the bond strength degradation characteristics, pull-out test results for concrete with RCA [23] or RFA [29] that are relevant to this study are listed in Table 7. As shown in Figure 6a, the maximum bond strength tended to decrease as the RCA*r* increased. As shown in Figure 6b, in the case of concrete with RFA-A, the bond strength did not seem to be affected by the RFA*r*, at least up to 60% RFA*r*. It is well known that RFA has higher water absorptivity values and lower specific gravity values than RCA. However, as shown in Figure 6c, the bond strength decreased as the RCA*r* increased, although the RFA*r* decreased.

It was noted that the RCA*r* was a much more dominant factor for the bond strength of concrete with RFCA than the RFA*r*, even though the bond strength of concrete with RCA or RFA was affected by each recycled-aggregate replacement level. In view of the bond-slip relationship for concrete with RFCA, it can be inferred that the effect of the RFA*r* was negligible because the effect of the RCA*r* on the bond strength of the RAC was by far the prevailing factor.

### 3.3. Casting Position Effect between Horizontal Rebars and RAC

When horizontal reinforcement is placed at over 300 mm from the bottom of the member, a decrease in the bond stress due to bleeding or sweating should be taken into account. In this study, tests on the pull-out specimens with horizontal bars at various depths (two- and three-tired) were conducted to examine the casting position effect on the bond strength of bar in RAC.

**Figure 7:**
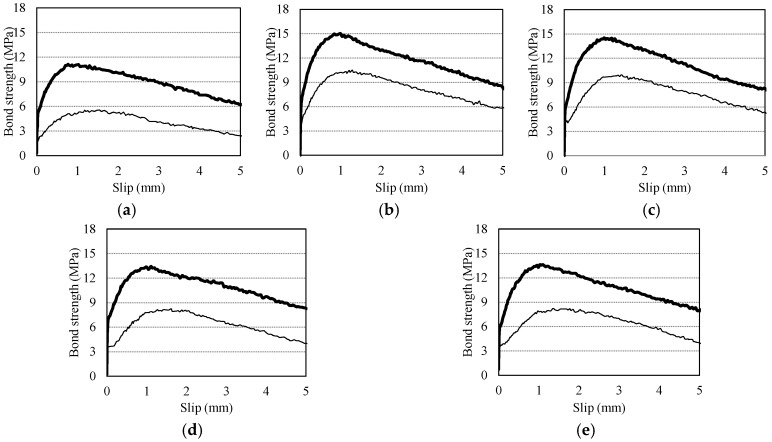
Bond strength-slip curves of two-tiered specimen. (**a**) G0F0H; (**b**) G15F60H; (**c**) G30F45H; (**d**) G45F30H; (**e**) G60F15H.

Figure 7 shows the bond strength-slip curves of the two-tiered pull-out specimens (75 mm, 225 mm in depth) in terms of mean curve. Data for the bottom and top specimens are plotted using thick and thin lines, respectively. The curves of the two-tiered pull-out specimens, which are similar to the V-type specimens, demonstrate that the maximum bond strength (*τ*_0_) tended to decrease and the slip at the maximum bond strength (*s*_0_) increased as the RCA*r* increased. This tendency can be seen in both the bottom and top specimens, although the normal concrete specimen G0F0H showed lower bond strength than the RAC specimens.

**Figure 8:**
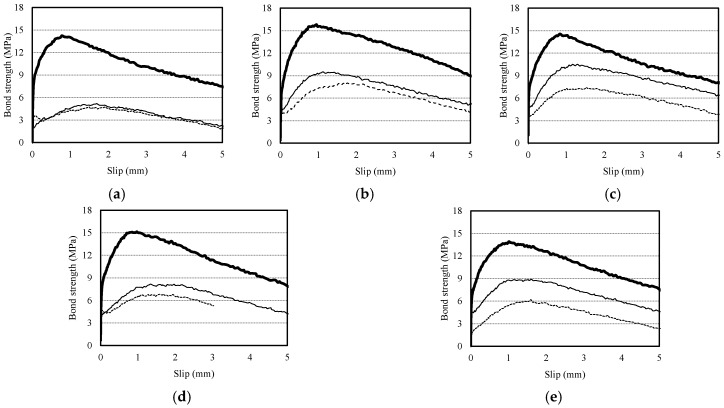
Bond strength-slip curve of three-tiered specimen. (**a**) G0F0R; (**b**) G15F60R; (**c**) G30F45R; (**d**) G45F30R; (**e**) G60F15R.

Figure 8 shows the bond strength-slip curves of the three-tiered pull-out specimens (75 mm, 225 mm, 375 mm in depth) in terms of mean value. The bottom, middle, and top specimens are plotted using thick lines, thin lines, and dotted thin lines, respectively. The curves of the three-tiered pull-out specimens, which are similar to those of the V- and H-type specimens, demonstrate that the maximum bond strength (*τ*_0_) tended to decrease and the slip at the maximum bond strength (*s*_0_) increased as the RCA*r* increased. This tendency was also observed in all the bottom, middle, and top specimens, similar again to the V- and H-type specimens. For the G0F0R specimen, the gap between the bond strength of the middle and top specimens was only 5.9%, which was negligible. For the RAC specimens, however, it is worth noting that the bond strength values of the middle and top specimens were closer to those of the bottom specimens compared to the G0F0R specimen.

**Figure 9:**
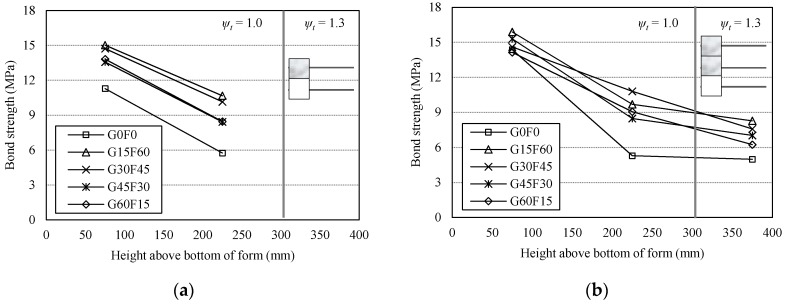
Comparison of bond strength levels in terms of location (height) of the rebar. (**a**) two-tiered specimens; (**b**) three-tiered specimen.

Figure 9 presents the bond strength levels of the two- and three-tiered specimens with different rebar location (height). As seen in Figure 9a, for G0F0 specimen, the bond strength of the top rebar specimens was 50.9% in comparison with the bottom rebar specimens. For the RAC specimens, the bond strength levels of the top rebar specimens were in the range of approximately 61.2% to 70.9% in comparison with the bottom rebar specimens. The bond strength ratios of the top to bottom specimens showed a drop as the RCA*r* increased, even though slight differences were observed among the bond strength levels of the RAC specimens. As presented in Figure 9b, for the middle rebar specimens (the RM series) of the RAC, the bond strength levels ranged from about 55.4% to 74.0% compared to the bottom rebar specimens (the RB series). These bond strengths of the middle rebar in the three-tiered specimens were similar to those of the top rebar in the two-tiered specimens. The top rebar specimens (the RT series) of the RAC showed bond strength levels in the range of approximately 44.2% to 52.1% of the bottom rebar specimens. The bond strength levels of the top rebar specimens ranged from approximately 69.0% to 85.4% of those of the middle rebar specimens.

**Figure 10:**
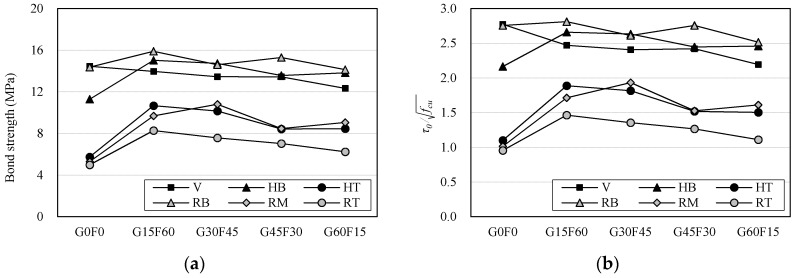
Comparison of bond strength levels. (**a**) Bond strength; (**b**) $\tau_{0}/\sqrt{f_{cu}}$.

Figure 10 compares the bond strength and normalized bond strength levels for all the pull-out test specimens. As presented in Figure 10a, the bond strength levels of the bottom specimens (HB and RB) were similar to those of the V-type specimens because the bond area located at the lower part of the specimens where the amount of settlement of the coarse aggregate was more than the settlement at the top part. The middle and top specimens (HT, RM, and RT) showed similar bond strength levels and deterioration tendencies as the RCA*r* increased. A similar tendency in terms of bond strength can also be observed in a previous study [23]. The bond between the RAC and the bar depends significantly on the mechanical anchorage resistance. It was noted that round-shaped RCA generally accelerated the settlement speed of the aggregate and increases the bleeding rate while the concrete was fresh. Thus, it is thought that the mechanical interlock between the rebar and the surrounding concrete in the top part was weakened. The bond strengths of the bar embedded in the top part were not over 2.5 (i.e., the good bond condition of the CEB-FIP model), as seen in Figure 10b. Furthermore, the normalized bond strength values of the G0F0RT, G45F30RT, and G60F15RT specimens were below 1.25, which was the other bond condition of the CEB-FIP.

**Figure 11:**
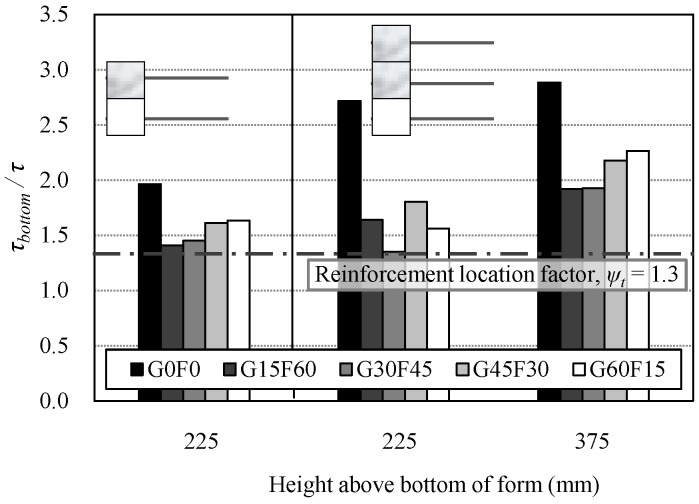
Casting position effect in terms of the location (height) of the rebar.

Figure 11 shows the location factors of the two- and three-tiered specimens. All the RAC specimens yielded location factors that were higher than those presented in ACI 318-14 for deformed steel rebar (1.3), even though both the H-type top rebar and the R-type middle rebar were placed in less than 300 mm of fresh concrete.

The pull-out test results of the two- and three-tiered specimens are summarized in Table 8 and Table 9, respectively. Based on the test results, it is noted that the predicted values from the ACI and CSA codes were significantly lower than the experimental results. Therefore, the mixtures of RAC used in this study could be applied to practical construction with ready-mix concrete because their bond strengths met the minimum bond strength for the development of the deformed rebar required by the ACI and CSA code provisions.

### 3.4. Analytical Predictions for Bond-Slip Relationship of RAC

For the analytical predictions of the bond-slip relationship of RAC, the following dimensionless bond strength ($\bar{\tau }$) and slip ($\bar{s}$) parameters of Xiao and Falkner [28] are used:

(2) $$\bar{\tau }=\frac{\tau }{\tau_{0}},\;\bar{s}=\frac{s}{s_{0}}$$

where $\tau_{0}$ is the peak bond strength, and $s_{0}$ is the slip corresponding to the $\tau_{0}$. Based on comparisons with the test results, the normalized bond-slip relationship of RAC can be expressed approximately as:

(3) $$\bar{\tau }=\{\begin{array}{c} {\left(\bar{s}\right)}^{a}\bar{s}\leq 1,\\ \frac{\bar{s}}{b{\left(\bar{s}-1\right)}^{2}+\bar{s}}\bar{s}>1 \end{array}$$

where *a* is a function of the slope of the ascending branch and b is related to the area under the descending branch of the stress-strain curve. Equation (3) was proposed by Haraji [39] and Guo [40] for normal concrete; in this study, Equation (3) was applied to RAC by modifying the *a* and *b* parameters.

**Figure 12:**
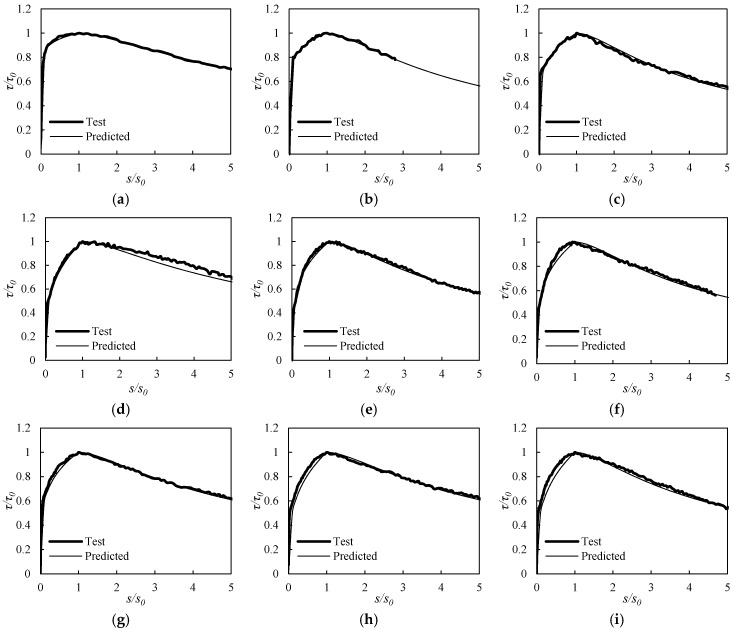

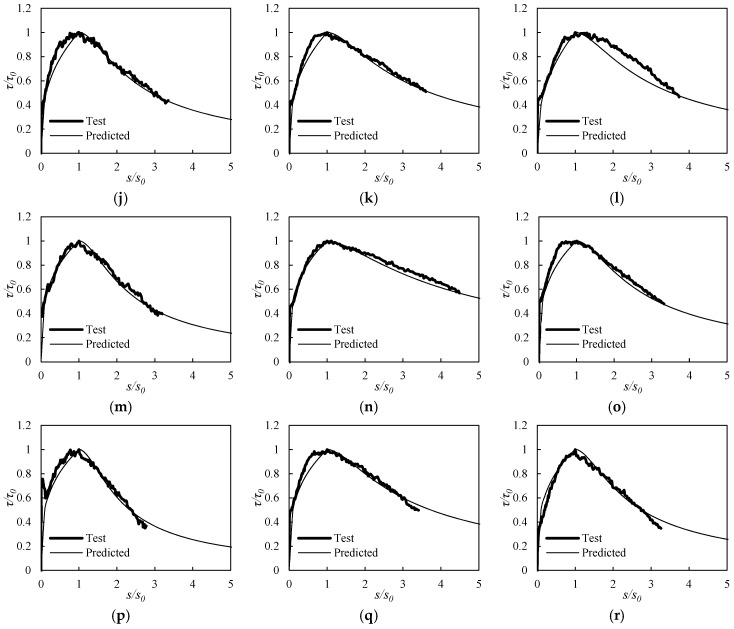
Typical comparisons of predicted bond behavior and test results. (**a**) G0F0V; (**b**) G30F45V; (**c**) G60F15V; (**d**) G0F0HB; (**e**) G30F45HB; (**f**) G60F15HB; (**g**) G0F0RB; (**h**) G30F45RB; (**i**) G60F15RB; (**j**) G0F0HT; (**k**) G30F45HT; (**l**) G60F15HT; (**m**) G0F0RM; (**n**) G30F45RM; (**o**) G60F15RM; (**p**) G0F0RT; (**q**) G30F45RT; (**r**) G60F15RT.

Figure 12 presents the averaged test curves and the curves predicted analytically using Equation (3) for typical pull-out test specimens. It can be seen that the test curves were closely predicted by Equation (3), which demonstrates that Equation (3) can be applied to express numerically the overall bond behavior of RAC with a bar.

Table 10 and Table 11 show the regression parameters *a* and *b*, respectively. For each recycled-aggregate replacement level, the value of parameter *a* for the ascending branch was found to fall within a narrow range and did not indicate any particular tendency. This trend of the effect of RCA*r* on *a* has been reported in other research [19,28].

**Figure 13:**
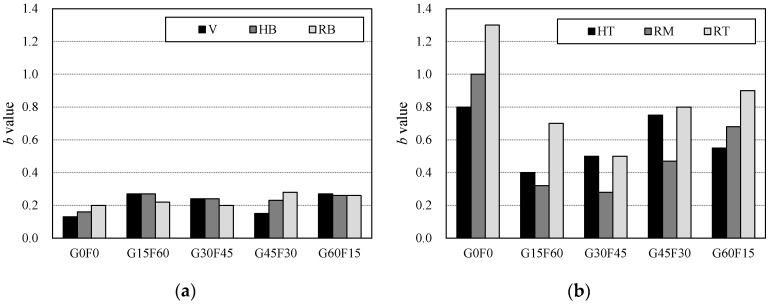
Comparison of b values. (**a**) Bar in lower part of member; (**b**) Bar in upper part of member.

Based on parameter *b* relative to the area under the descending branch, provided that the bar is located in the lower part of the concrete member, as shown in Figure 13a, it is found that RAC specimens exhibit similar post-peak energy-absorbing capacities to normal concrete (G0F0 specimen). However, in the case of the bar being located in the upper part of the member, it can be concluded from the variation of the *b*-values presented in Figure 13b that the energy absorption capacity of the RAC is higher than that of the normal concrete. It can also be seen that the energy absorption capacity decreases as the RCA replacement increases.

## 4. Conclusions

The following observations and conclusions were drawn on the basis of pull-out test results in this study.

(1) The maximum bond strength (*τ*_0_) tends to decrease and the slip at the maximum bond strength (*s*_0_) increases as the recycled coarse aggregate replacement level increases, irrespective of the rebar orientation and placement location.
(2) For the effect of recycled-coarse aggregate on the bond strength, water absorption of the coarse aggregate is more considerable than their replacement level.
(3) In case of the three-tiered specimens, the bond strength levels at the middle height (225 mm depth) are similar to those of the top rebar in case of the two-tiered specimens. Therefore it can be inferred that the top bar effect can be found from the mid-height of a member, irrespective of the member’s height.
(4) It can be concluded that the recycled aggregate concrete mixtures used in this study could be used in actual construction practices that employ ready-mixed concrete.

## Figures and Tables

**Table 1:** Physical characteristics of aggregate used in this study.

Aggregate	Maximum Grain Size (mm)	Density (g/cm^3^)	Water Absorption (%)	Fineness Modulus
NCA	25	2.68	0.59	6.66
RCA	25	2.51	1.84	5.40
NFA	5	2.46	2.70	2.53
RFA	5	2.35	4.43	2.99

**Table 2:** Mix proportions of concrete samples.

Mix.	Recycled-Aggregate Replacement Level (%)		W/C	S/a	Unit Weight (kg/m^3^)						
	RCA	RFA			W	C	NCA	RCA	NFA	RFA	AD
G0F0	0	0	0.41	0.46	180	435	884	0	730	0	2.18
G15F60	15	60			180	435	790	133	307	438	2.18
G30F45	30	45			177	435	650	265	422	328	2.18
G45F30	45	30			177	435	511	398	538	219	2.18
G60F15	60	15			177	435	372	530	653	109	2.18

Note: W/C is water-to-cement ratio; S/a is sand-to-aggregate ratio; W is water; C is cement; and AD is admixture.

**Table 3:** Description of test specimens.

Specimen	*f_ck_* (MPa)	Rebar Orientation and Placement					
		V	HB	HT	RB	RM	RT
G0F0	27	3	3	3	3	3	3
G15F60		3	3	3	3	3	3
G30F45		3	3	3	3	3	3
G45F30		3	3	3	3	3	3
G60F15		3	3	3	3	3	3

Note: *f_ck_* is design strength of concrete; G is recycled coarse aggregate (i.e., gravel); F is recycled fine aggregate (i.e., sand). Recycled-aggregate replacement level: 0%, 15%, 30%, 45%, or 60%. Rebar orientation: V is vertical; H is horizontal two-tier; R is horizontal three-tier. Rebar placement: T is top; M is middle; B is bottom.

**Table 4:** Mechanical properties of concrete at 28-day.

Specimen	*f_cu_* (MPa)	*ε_cu_* (×10^−6^)	*E_c_* (GPa)	*f_sp_* (MPa)	*λ*
G0F0	27.2	1905	24.7	2.04	0.70
G15F60	32.0	1937	23.8	2.50	0.80
G30F45	31.3	1942	24.9	2.33	0.75
G45F30	30.8	2065	23.4	2.48	0.80
G60F15	31.6	2002	23.8	2.31	0.74

Note: *f_cu_* is 28-day compressive strength; *ε_cu_* is strain at peak strength; *E_c_* is modulus of elasticity; *f_sp_* is splitting tensile strength; *λ* is modification factor reflecting the reduced mechanical properties of concrete.

**Table 5:** Bond strength of V-type specimens at 28 days.

Specimen	*τ*_0_ (MPa)	*s*_0_ (mm)	$\tau /\sqrt{f_{cu}}$	S.D. ^1^ (MPa)	C.V. ^2^ (%)	Calculated Bond Strength (MPa)		
						Orangun	Darwin	CEB-FIP
G0F0	14.46	0.48	2.77	1.23	8.54	11.37	12.42	13.03
G15F60	13.97	0.84	2.47	1.18	8.42	12.33	13.47	14.13
G30F45	13.46	0.63	2.41	2.06	15.28	12.20	13.32	13.98
G45F30	13.44	0.43	2.42	1.71	12.72	12.10	13.22	13.87
G60F15	12.34	0.69	2.22	0.47	3.82	12.27	13.41	14.07

^1^ Standard Deviation, ^2^ Coefficient of Variation.

**Table 6:** Parameters for defining mean bond strength (CEB-FIP model code, 1990).

Unconfined Concrete ^1^		Confined Concrete ^2^	
Good Bond Conditions	All Other Bond Conditions	Good Bond Conditions	All Other Bond Conditions
$2.0\sqrt{f_{ck}}$	$1.0\sqrt{f_{ck}}$	$2.5\sqrt{f_{ck}}$	$1.25\sqrt{f_{ck}}$

^1^ Failure by splitting of the concrete. ^2^ Failure by shearing of the concrete between the ribs.

**Table 7:** Physical properties of recycled aggregate and RAC in other studies.

Concrete Type	Maximum Grain Size (mm)		Specific Gravity			Water Absorption (%)			$f_{c}^{\prime }$ (MPa)
	Coarse	Fine	RCA	RFA-A	RFA-B	RCA	RFA-A	RFA-B	
RCA (Jau) [19]	19	5	2.29	-	-	5.26	-	-	21–35
RCA (Kim) [23]	25	5	2.48	-	-	3.01	-	-	29–33
RFA (Kim) [29]	25	5	-	2.29	2.15	-	5.83	7.95	27–32

**Table 8:** Bond strengths of H-type specimens.

Specimen	$f_{c}^{\prime }$ (MPa)	Location	$\tau_{0}$ (MPa)	Location Factor	Current Code (MPa)	
					ACI	CSA
G0F0H	27.18	Bottom	11.29		2.71	3.39
		Top	5.75	1.96	2.08	2.60
G15F60H	31.95	Bottom	15.04		2.94	3.67
		Top	10.67	1.41	2.26	2.82
G30F45H	31.28	Bottom	14.74		2.91	3.63
		Top	10.16	1.45	2.24	2.79
G45F30H	30.79	Bottom	13.58		2.88	3.60
		Top	8.43	1.61	2.22	2.77
G60F15H	31.66	Bottom	13.83		2.92	3.65
		Top	8.46	1.63	2.25	2.81

**Table 9:** Bond strengths of R-type specimens.

Specimen	$f_{c}^{\prime }$ (MPa)	Location	$\tau_{0}$ (MPa)	Location Factor	Current Code (MPa)	
					ACI	CSA
G0F0R	27.18	Bottom	14.38		2.71	3.39
		Middle	5.29	2.72		
		Top	4.98	2.89	2.08	2.60
G15F60R	31.95	Bottom	15.90		2.94	3.67
		Middle	9.69	1.64		
		Top	8.28	1.92	2.26	2.82
G30F45R	31.28	Bottom	14.61		2.91	3.63
		Middle	10.81	1.35		
		Top	7.58	1.93	2.24	2.79
G45F30R	30.79	Bottom	15.30		2.88	3.60
		Middle	8.48	1.81		
		Top	7.03	2.18	2.22	2.77
G60F15R	31.66	Bottom	14.14		2.92	3.65
		Middle	9.06	1.56		
		Top	6.25	2.26	2.25	2.81

**Table 10:** Regression Parameter *a*.

Specimen	V	HB	HT	RB	RM	RT
G0F0	0.07	0.30	0.35	0.23	0.30	0.30
G15F60	0.18	0.23	0.35	0.30	0.30	0.30
G30F45	0.12	0.26	0.35	0.30	0.30	0.30
G45F30	0.09	0.22	0.40	0.30	0.30	0.30
G60F15	0.18	0.26	0.40	0.30	0.30	0.30

**Table 11:** Regression Parameter *b*.

Specimen	V	HB	HT	RB	RM	RT
G0F0	0.13	0.16	0.80	0.20	1.00	1.30
G15F60	0.27	0.27	0.40	0.22	0.32	0.70
G30F45	0.24	0.24	0.50	0.20	0.28	0.50
G45F30	0.15	0.23	0.75	0.28	0.47	0.80
G60F15	0.27	0.26	0.55	0.26	0.68	0.90
